# Interdomain Linker Effect on the Mechanical Stability of Ig Domains in Titin

**DOI:** 10.3390/ijms23179836

**Published:** 2022-08-30

**Authors:** Bei Tong, Fang Tian, Peng Zheng

**Affiliations:** 1 Institute of Botany, Jiangsu Province and Chinese Academy of Sciences, Nanjing 210014, China; 2 State Key Laboratory of Coordination Chemistry, Chemistry and Biomedicine Innovation Center (ChemBIC), School of Chemistry and Chemical Engineering, Nanjing University, Nanjing 210023, China

**Keywords:** titin, force spectroscopy, interdomain linker

## Abstract

Titin is the largest protein in humans, composed of more than one hundred immunoglobulin (Ig) domains, and plays a critical role in muscle’s passive elasticity. Thus, the molecular design of this giant polyprotein is responsible for its mechanical function. Interestingly, most of these Ig domains are connected directly with very few interdomain residues/linker, which suggests such a design is necessary for its mechanical stability. To understand this design, we chose six representative Ig domains in titin and added nine glycine residues (9G) as an artificial interdomain linker between these Ig domains. We measured their mechanical stabilities using atomic force microscopy-based single-molecule force spectroscopy (AFM-SMFS) and compared them to the natural sequence. The AFM results showed that the linker affected the mechanical stability of Ig domains. The linker mostly reduces its mechanical stability to a moderate extent, but the opposite situation can happen. Thus, this effect is very complex and may depend on each particular domain’s property.

## 1. Introduction

**Figure 1:**
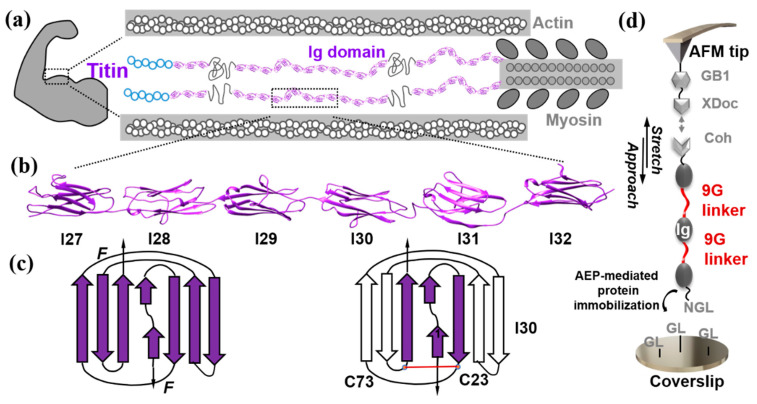
(**a**) Schematic architecture of one-half of the sarcomere highlights the polyprotein structure of giant protein titin (colored in purple), not in scale. (**b**) Structure shows human titin segment I(27–32) chosen for mechanical stability measurements. Except for I27 (PDB:1TIT), all others are simulated structures. (**c**) The cartoon shows the structure of I30 with a possible disulfide bond between Cys73 and Cys23, while no disulfide is present in other Ig domains. (**d**) The schematic shows how high-precision AFM-SMFS measures the mechanical stability of Ig domains with an artificial linker.

The giant muscle protein titin is a tandem modular construction designed polyprotein containing more than two hundred individually folded domains, such as immunoglobulin-like (Ig) and fibronectin-type III domains [1]. These domains are similar in size (~2 nm) and length (~90 residues). Interestingly, these domains are closely connected with very few residues in-between. Functionally, the I-band part of titin is extensible and plays a critical role in the passive elastic properties of muscles (Figure 1a). Thus, the molecular design for such a giant polyprotein is of great interest in understanding its mechanical function [2,3,4,5]. Here, we are particularly interested in whether the interdomain amino acid sequence/linker affects the mechanical stability of the Ig domains in titin, which has been studied with great interest [6,7,8].

To examine the linker effect, we used atomic force microscopy-based single-molecule force spectroscopy (AFM-SMFS) to measure the mechanical stability of human Ig domains. SMFS can manipulate a single molecule mechanically [9,10,11,12,13,14,15,16,17,18,19,20,21,22] and AFM-SMFS has been widely used to study the mechanical stability of proteins [23,24,25,26,27,28,29], protein-protein interactions [30,31,32,33,34,35,36,37,38,39,40,41,42,43], and chemical bonds [44,45,46,47,48,49,50,51,52,53,54,55,56]. Many titin domains have been studied [57,58,59]. For example, I27, the 27th Ig domain in titin (also called I91 based on a different nomenclature), is one of the first and most studied protein domains by AFM-SMFS, showing a force of ~200 pN and a contour length increment (ΔLc) of 28 nm upon unfolding [60]. In addition, the effect of the disulfide bond in I27 has been studied in detail [61,62]. 

Thus, we choose six consecutive Ig domains of titin, including I27, I28, I29, I30, I31, and I32, as a representative unit (I27–I31), to study the interdomain linker effect (Figure 1b). The structures and mechanical stabilities of many of these domains have been well determined. The crystal structure of I27 is available, while structures for other domains are constructed by the I-TASSER server (Figure 1b). It is noted that I27 is a historical nomenclature, especially in the AFM-SMFS field, and we used it here. It is renamed I91 later [63]. There are 89 extensible residues in each Ig domain except for I30, leading to ΔLc of ~28 nm, as shown for I27. For I30, a disulfide bond may be formed between the Cys23 and Cys73, leading to a smaller ΔLc with only 51 extensible residues (Figure 1c). In addition, we designed nine glycines (9G) as an artificial linker due to its simple structure without a self-secondary structure.

## 2. Results

**Figure 2:**
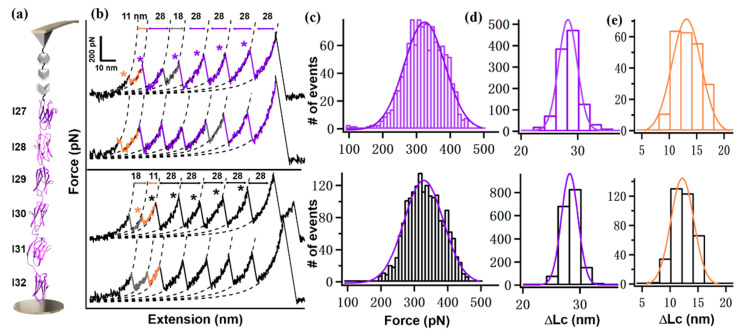
(**a**) Scheme of AFM-SMFS setup to measure the mechanical stability of polyprotein I(27–32). (**b**) Representative force-extension curves show the unfolding events of Ig domains (marked by a star). The top two curves are from the unfolding of polyprotein with an artificial 9G linker (colored in purple) and the bottom curves are from the natural polyprotein sequence without the linker. The I30 domain shows a peak with ΔLc of 11 nm (in orange), the remaining five Ig domains all show a peak with ΔLc of 28 nm, and GB1 shows a ΔLc of 18 nm. (**c**,**d**) Histograms show the unfolding force (**c**) and ΔLc (**d**) from the five Ig domains. (**e**) The histogram shows the ΔLc of I30.

A high-precision AFM measurement system has been used for accurate measurement and comparison of Ig domains in titin [64,65,66,67] (Figure 1d). In short, the target polyprotein designed with a specific peptide sequence NGL was immobilized on a GL peptide-coated surface through AEP (asparaginyl endopeptidase)-mediated protein ligation between the two peptide sequences [68]. A GB1-XDoc coated-AFM tip was used to probe the target polyprotein (Figure 2c). Here, GB1 with known properties (Force = 180 pN, ΔLc = 18 nm) was added, serving as an internal force caliper [69]. The reversible protein-protein interaction Cohesion:XDockerin (Coh:XDoc) was used to enable efficient protein pick-up [64].

Thus, we built polyprotein Coh-I(27–32)/9G-NGL with a 9G linker for measurement. The 9G linker is present between each Ig domain except for the two end I27 and I32 domains (Figure 2a). By approaching the AFM tip towards the surface, the polyprotein was picked up between the Coh:XDoc interaction. Upon stretching, the polyprotein was under mechanical manipulation and its corresponding force-extension curve showed characteristic sawtooth-like peaks from the stepwise unfolding of each domain, and a final rupture peak with a higher force of ~600 pN was observed from the break of Coh:XDoc complex. By fitting the elasticity of the curve using the worm-like chain model [70], five unfolding events from Ig domains were obtained, showing a ΔLc of 28 ± 2 nm (Figure 2b), agreeing well with the theoretical value. Moreover, the unfolding forces of different Ig domains are similar. The force histogram showed a single peak with an average unfolding force of 308 ± 64 pN (ave. + stdv. *n* = 1148, Figure 2c). Besides, an additional peak with a ΔLc of 11 ± 2 nm was observed, which was from the partial unfolding of I30 (Figure 1c). As described, a disulfide bond is indeed presented in I30. Thus, only 40 residues can be unfolded, leading to a theoretical value of 10.4 nm (40 × 0.36 − 4.0 nm).

Then, we used polyprotein Coh-I(27–32)-NGL with natural sequence for AFM measurement and comparison. The same cantilever used previously for the polyprotein with the linker was used here again to minimize the error. As expected, a similar unfolding pattern was observed (Figure 2b,d,e). However, the force is slightly lower, with a value of 324 ± 54 pN (*n* = 1808, Figure 2c) (Table 1). 

**Figure 3:**
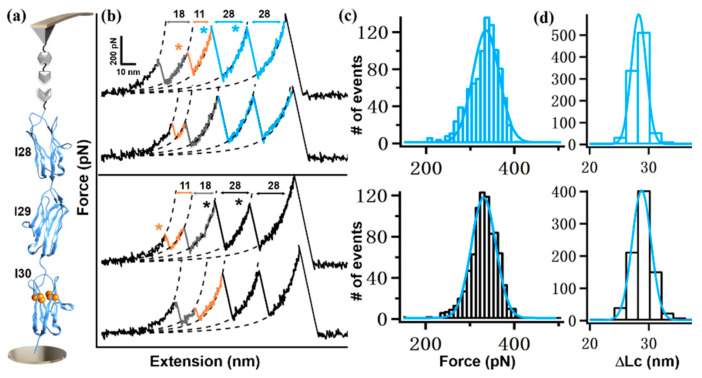
(**a**) Scheme of AFM-SMFS setup to measure I(28–30) domains. (**b**) Representative force-extension curves from polyprotein with linkers (top, colored in blue) and without linker (bottom, black). (**c**,**d**) Histograms of their corresponding unfolding force (**c**) and ΔLc (**d**) are shown. The star indicates the unfolding event/peak of one protein domain.

**Figure 4:**
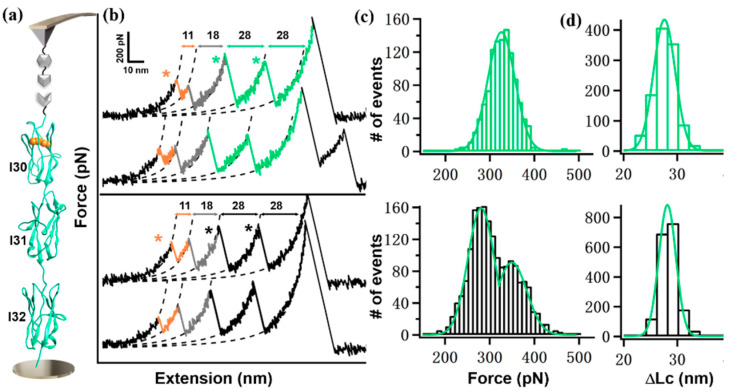
(**a**) Scheme of AFM-SMFS setup to measure I(30–32) domains. (**b**) Representative force-extension curves from polyprotein with linkers (top, colored in green) and without linker (bottom, in black). (**c**,**d**) Histograms of their corresponding unfolding force (**c**) and ΔLc (**d**) are shown. The star indicates the unfolding event/peak.

To confirm this effect, we chose three Ig domains only and constructed two shorter polyproteins, Coh-I(28–30)/9G-NGL and Coh-I(30–32)/9G-NGL, for measurement. Indeed, stretching these polyproteins resulted in a shorter force-extension curve with only two 28 nm-peaks from I28, I29, or I31, I32, and one 11 nm-peak from I30, as expected (Figure 3 and Figure 4). For Coh-I(28–30)/9G-NGL, the histogram of unfolding forces from I28 and I29 showed a single peak with an average force of 330±36 pN (*n* = 860). Then, AFM measurement on Coh-I(28–30)-NGL with natural sequence showed a force of 325 ± 35 pN (*n* = 965).

For Coh-I(30–32)/9G-NGL, the histogram of unfolding force from I31 and I32 showed a single peak with an average force of 320 ± 33 pN (*n* = 1120). However, AFM measurement on the natural sequence showed a different result. Two peaks were observed in the histogram, with a force of 276 pN and 345 pN, respectively (*n* = 1804), which has not been observed before. 

As a result, the unfolding force of Ig domains in titin is generally lower when the 9-glycine length amino acids sequence is present as an interdomain linker. Moreover, the effect can be complex when considering the unfolding force of I31 and I32. 

**Figure 5:**
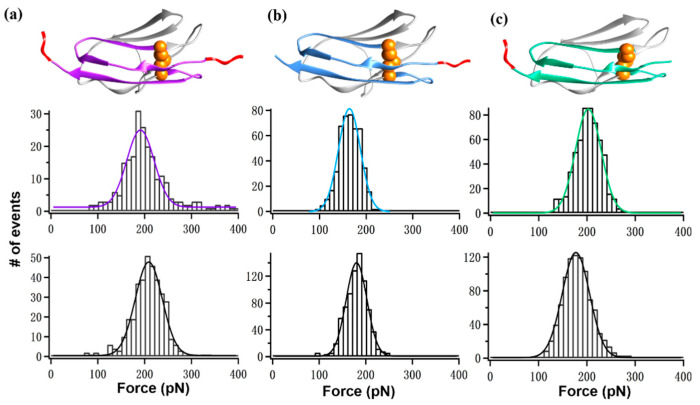
(**a**–**c**) The scheme (top panel) and mechanical force of I30 in polyprotein I(27–32) (**a**), I(28–30) (**b**), and I(30–32) (**c**). The unfolding force histogram of I30 with the corresponding linker is shown in the middle panel, and the natural sequence without linker is shown at the bottom. The 9G linker was colored red, and the two cysteines forming the disulfide bond were depicted as orange spheres.

Finally, we focused on the unfolding results of I30 in each polyprotein design. First, with an internal disulfide bond, I30 showed a unique 11 nm-peak which can be distinguished from other Ig domains. Thus, its unfolding force can be assigned unambiguously. Moreover, the linker situation for I30 in the three polyproteins is different (Figure 5). For I(27–32), 9G linker is present on both sides of I30 (Figure 5a). In this design, the unfolding force of I30 was 203 ± 34 pN (*n* = 360), and 186 ± 55 pN (*n* = 225) without linker. For I(28–30), 9G linker is only present on the N terminus. The force was 197 pN (*n* = 570), and 172 ± 24 pN (*n* = 910) without linker. Finally, for I(30–32), 9G linker is only present on the C terminus. The force was 159 ± 23 pN (*n* = 477), and 175 ± 24 pN (*n* = 404) without the linker (Table 2).

Based on these results for I30, we found that this linker effect is much more complex than we thought before. Indeed, a few cases have been studied for the linker effect, both mechanically and thermodynamically [71,72]. No general trend/conclusion has been obtained. In this work, we found the linker can reduce the mechanical stability of I30 when present in I(27–32) and I(30–32) while increasing it when present in I(28–30). Nevertheless, it is no doubt that the linker affects the domain stability in titin.

In this work, we determined the effect of the interdomain linker for the Ig domain in titin. By measuring the mechanical stability of polyprotein containing multiple Ig domains in titin, with/without an artificial 9G linker, we found the linker indeed affects the Ig domain’s stability. The force is reduced when an artificial linker is present in most cases. However, the extent can vary; sometimes the trend is reversed. Thus, we believe this linker effect is much more complex, and the intrinsic property of each domain and the linker itself play important roles. Nevertheless, this work provides a glimpse of the molecular design of the giant titin, and future studies are needed to understand this important molecule for humans and even for designing artificial muscle [73,74,75].

## 3. Method and Material

**Protein engineering:** The plasmid: Coh-I(27–32)/9G-NGL, Coh-I(27–32)-NGL, Coh-I(28–30)/9G-NGL, Coh-I(28–30)-NGL, Coh-I(30–32)/9G-NGL, Coh-I(30–32)-NGL were obtained after Gibson assembly-based method [76]. All plasmids were overexpressed in *Escherichia coli* strain BL21 (DE3) and cells were cultured overnight in LB medium at 18 °C by the addition of 1mM IPTG. The cells were pelleted by centrifugation and the polyprotein purification by Ni-NTA affinity. After using wash buffer (50 mM Tris, 100 mM NaCl, 20 mM imidazole, pH 7.4) to purify the target proteins, the polyproteins were eluted in elution buffer (50 mM Tris, 100 mM NaCl, 200 mM imidazole, pH 7.4). Protein ligase AEP was obtained according to literature [77].

**Protein immobilization:** The glass coverslips (Sail Brand, China) and probes (MLCT-Bio-DC, Bruker) were cleaned by plasma. Then, both probes and coverslips were immersed in 1% (*v/v*) APTES toluene solution for 1 h to add the NH_2_ group, followed by a reaction with Milli-Q water containing 2 mM ImSO_2_N_3_, 4 mM K_2_CO_3_, and 20 mM CuSO_4_ to add the N_3_ group. After flushing, they were further reacted with DBCO-PEG_4_-maleimide for at least 2 h to add the maleimide group. Peptide C-ELP_20_-NGL and GL-ELP_20_-C were respectively reacted onto the probe and coverslip. For AFM-SMFS measurement, 50 μL AFM buffer (100 mM Tris, 100 mM NaCl, pH 7.4) containing 100 μM Ig proteins and 50 μM AEP were pipetted on the glass slides for 40 min. The cantilevers were incubated with 50 μL solution of 60 μM GL-CBM-XDoc and 50 Μm AEP in the AFM buffer.

**AFM-SMFS Experiment:** Measurements using Coh-Doc interaction of high rupture force as a standard were carried out Nanowizard4 (JPK) atomic force microscope. Using the equipartition theorem, the spring constant of ~30 pN nm^−1^ was obtained by calibrating the MLCT-Bio-DC (Bruker) cantilever in the AFM buffer solution. The functionalized cantilevers and glass coverslip immobilize polyproteins in AFM buffer at pH 7.4. All AFM experiments were performed at a constant pulling speed of 1000 nm·s^−1^.

Protein sequence (I27-**9G**-I28-**9G**-I29-**9G**-I30-**9G**-I31-**9G**-I32)

LIEVEKPLYGVEVFVGETAHFEIELSEPDVHGQWKLKGQPLAASPDCEIIEDGKKHILILHNCQLGMTGEVSFQAANTKSAANLKVKEL**GGGGGGGGG**PLIFITPLSDVKVFEKDEAKFECEVSREPKTFRWLKGTQEITGDDRFELIKDGTKHSMVIKSAAFEDEAKYMFEAEDKHTSGKLIIEGI**GGGGGGGGG**RLKFLTPLKDVTAKEKESAVFTVELSHDNIRVKWFKNDQRLHTTRSVSMQDEGKTHSITFKDLSIDDTSQIRVEAMGMSSEAKLTVLEG**GGGGGGGGG**DPYFTGKLQDYTGVEKDEVILQCEISKADAPVKWFKDGKEIKPSKNAVIKTDGKKRMLILKKALKSDIGQYTCDCGTDKTSGKLDIEDR**GGGGGGGGG**EIKLVRPLHSVEVMETETARFETEISEDDIHANWKLKGEALLQTPDCEIKEEGKIHSLVLHNCRLDQTGGVDFQAANVKSSAHLRVKPR**GGGGGGGGG**VIGLLRPLKDVTVTAGETATFDCELSYEDIPVEWYLKGKKLEPSDKVVPRSEGKVHTLTLRDVKLEDAGEVQLTAKDFKTHANLFVKEP

## Figures and Tables

**Table 1:** Unfolding force of Ig domains for each polyprotein design.

Polyprotein	9G Linker	No Linker
I(27–32)	308 ± 64 pN (*n* = 1148)	324 ± 54 pN (*n* = 1808)
I(28–30)	325 ± 35 pN (*n* = 965)	330 ± 36 pN (*n* = 860)
I(30–32)	320 ± 33 pN (*n* = 1120)	276 pN/345 pN (*n* = 1804)

**Table 2:** Unfolding force of I30 in each polyprotein design.

Polyprotein	9G Linker	No Linker
I(27–32)	186 ± 55 pN (*n* = 225)	203 ± 34 pN (*n* = 360)
I(28–30)	159 ± 23 pN (*n* = 477)	175 ± 24 pN (*n* = 404)
I(30–32)	197 ± 26 pN (*n* = 570)	172 ± 30 pN (*n* = 910)

## Data Availability

Data is contained within the article.
